# Marginal bone resorption of posterior mandible dental implants with different insertion methods

**DOI:** 10.1186/s12903-020-1019-7

**Published:** 2020-01-31

**Authors:** Ehsan Aliabadi, Saeid Tavanafar, Mohammad Saleh Khaghaninejad

**Affiliations:** Department of Oral and Maxillofacial Surgery, School of Dentistry, Shiraz University of Medical Sciences, Shiraz, Iran

**Keywords:** Dental implant, Bone loss, Implant fixture, Torque devices

## Abstract

**Background:**

To evaluated the marginal bone loss around dental implants by two insertion methods.

**Methods:**

Eligible patients were divided into two groups; manual and mechanized groups. Peri-apical x-ray using a customized device to standardize the radiographs designed and used to take three periodical radiographs; after surgery, three months, and six months follow up. An independent t-test was used to compare the two groups regarding the average level of marginal bone loss (*p* < 0.05).

**Results:**

After excluding dropouts, a total of 273 patients (120 males and 153 females, aged between 25 and 67 years old) were included in the study. The average marginal bone loss in the manual insertion method was 0.44 ± 0.84 mm, and 0.59 ± 0.20 mm, and for the mechanized method was 0.51 ± 0.20 mm and 0.67 ± 0.19 mm after three and six months, respectively. There was a significant difference in marginal bone loss after six months between the two groups(*p* < 0.001). However, no differences were observed after three months (*p* = 0.24).

**Conclusions:**

Under the condition of this study, both techniques were safe and resulted in an acceptable amount of bone resorption; however, in the manual method, the less marginal bone loss occurred after six months.

## Background

Osseo-integrated dental implants have demonstrated long-time success in the reconstruction of fully and partially edentulous patients [1]. Recently, a dental implant was considered the first treatment choice for prosthetic rehabilitation [2]. A dental implant is a prosthetic or alloplastic material, which is embedded in the bone, to maintain retention and support for fixed or removable dental prosthetics.

The treatment outcome depends on the trabecular and medullary bone structures and the level of bone resorption in the dental socket [3]. Various implant systems and prosthetics have been available for dentists and laboratory technicians during recent decades [4]. The variety of surgical methods in different systems has relatively disoriented the dentists to system selection. The modern implants are used to provide function, aesthetic, and durability compatible with natural teeth, besides successful implant insertion [5].

One of the challenging obstacles in dental implant treatment is marginal bone resorption. The cause is considered to be multifactorial, and heat generation might be one of the reasons [6]. Overheating the implant insertion area generally leads to thermal damage in bone tissues. All the drilling and insertion procedures damage the bone and destroy the implant stability, and mobility [7]. Manufacturers have introduced manual and mechanized implant insertions for the importing and hardening of implant screws [8].

In the manual method, the implant is inserted manually by using a screwdriver and ratchet. In this method, one hand grasps the ratchet, the thumb is positioned on the ratchet center of the rotation, the index finger retracts the lip, and the middle finger is positioned under the jaw along the path of osteotomy [9].

The mechanized method benefits from a drill or handpiece, which has a specific connector for holding the implant. A light apical pressure inserts the implant in the site of osteotomy [10].

Applying excessive pressure increases the bone necrosis and formation of non-vital bone during the healing stage [11]. Ratchets and manual drivers are more comfortable to use, cheaper, and transfer the tactile sense better [12]. It was reported that ratchets are preferred in D1 bones with higher density because the use of mechanized drivers and handpieces is accompanied by a higher risk of unnecessary friction and handpiece fracture [13].

Mechanized drivers can better control the insertion speed and torque of the fixture. Implant manufacturers mostly recommend mechanized drivers and handpieces for reaching the appropriate torque in clinical procedures [14]. Handpieces are better for implant insertion in the bones of lower density like D2 and D3. They provide the maximum required insertion force better [15, 16].

Implant insertion is not an easy task, especially in the posterior region of the ridges since different techniques have been invented to facilitate the importing of the implant to the bone with adequate torque [17]. Today, different implant systems use variable ratchets and drills to implant insertion, retain the screws, and healing abutments and other parts [18].

The stability of the surrounding tissues is considered a fundamental factor in the outcome of the implant, which is measured with radiologic standards [19]. Marginal bone resorption plays an imperative role in the stability of the mechanical implant [20], i.e., the more bone is around the implant, the better the stability, hygiene, and aesthetic outcome will be achieved [21].

Radiographic evaluations are a very renowned way to assess the longitudinal bone attachment to the implant [22]. The little amount of bone resorption around the implant is considered normal [23, 24]. The amount of bone resorption in the first year of implant function is about 0.9–1.6 mm. Moreover, the average bone resorption of 0.05–0.13 mm annually in the following years is considered normal [25]. Initial bone resorption diagnosis is essential because it gives the clinician a guideline for the necessity of corrective and preventive treatments [26].

To the best of our knowledge, no study has compared the outcomes of manual and mechanized dental implant fixture insertion methods. The present study aimed to survey the level of marginal bone resorption around the implants inserted either with manual or mechanized insertion techniques in patients treated for posterior mandible dental implants.

## Methods

### Patient population

The ethical committee of Shiraz University of Medical Sciences, Shiraz, Iran, has reviewed and approved this research study (approval ID: IR.SUMS.REC.1398.475) and guidelines of the Helsinki Declaration were followed. In the present study, all of the patients (*n* = 273) who attended our maxillofacial clinic for posterior mandibular implants between 2017 to 2019 after considering inclusion criteria were asked to participate in the study. However, power analysis showed acceptable power value for comparing the two groups after 6 months (power > 80%). Power analysis was done just in the 6 months because the differences and the amount of standard deviation were rational (effect size was sensible to conduct power analysis). All of the procedures were explained to each participant in a quiet room before their recruitment in the study, and patients were asked to sign a written informed consent. All of the surgical procedures were performed by the same experienced oral and maxillofacial surgeon. An experienced prosthodontist has completed the prosthetic procedures for all of the participants.

### Inclusion and exclusion criteria

Inclusion criteria were candidates of implant treatment in the mandibular molar and premolars area of any age, Angle class 1 occlusion (mesiobuccal cusp of the first maxillary molar in the buccal groove of the first mandibular molar), opposed natural dentition or fixed prosthesis, bone quality of D2 or D3, determined either radiographically or during the surgical insertion of implant by surgeon, and those required implant size of 11.5 × 4.5 mm (height×diameter).

Exclusion criteria were implant inserted for an overdenture, class 2 and 3 angle classification of malocclusions, patients with heavy bruxism and clenching, masticatory muscle hypertrophy, temporomandibular diseases, facial growth abnormalities (cleft lip and palate, hemifacial microsomia), psychological disorders, under treatment with antiresorptive drugs, bone qualities of D1 and D4, fixture exposure due to insufficient bone width during the surgical process and need for guided bone regeneration, infection of implant site, presence of partial or absolute contra-indication for implant treatment, such as uncontrolled metabolic diseases (diabetes, osteoporosis), recent episodes of radio- and chemotherapy and bone disease (osteomalacia, Paget’s disease), periodontal complications and poor oral hygiene before the surgery (grade 2 or 3, Loe and Silness gingival index and plaque index> 15% according to O’Leary index) [27], less than 2 mm attached gingival thickness buccolingual, heavy tobacco users, and the patients who needed guided bone regeneration around their dental implants.

### Implant site preparations and insertion methods

The dental implants used were UFII (DIO Co., Busan, Korea) endosteal root-form dental implants. The surgery was performed following the manufacturer’s instructions. All patients were given 2 g amoxicillin prophylaxis (Amoxicillin 500 mg; KosarDaru, Tehran) 1 h preoperatively, and they were asked to rinse their mouth with 0.2% chlorhexidine mouthwash (Chlorhexidine SHD 0.2%, Behsa, Tehran, Iran) for 1 min.

The site was anesthetized using infra-alveolar nerve block and infiltration with 2% lidocaine and 1:80000 epinephrine (Persocaine-E, Darou Pakhsh Mfg. Co., Tehran, Iran). Mid-crestal incision was performed on the alveolar ridge, and full-thickness mucoperiosteal flap reflected, and the alveolar bone was exposed. A round surgical bur used to create a flat bony surface, then, implant site preparation procedures started using sequential manufacturer drills. Copious irrigation with normal saline used to prevent overheating during the drilling procedure. Implant fixtures were randomly inserted into the osteotomy site either by manual or mechanized at the crestal level.

In the manual insertion group, the fixture was removed from the box by manual and inserted using a ratchet connected to a torque wrench. The ratchet was grasped with one hand, the thumb on the center of the rotation of the ratchet, the middle finger under the jaw along the path of the osteotomy, and the index finger retracted the lip. The torque wrench was used to adjust the amount of applied torque, keeping the maximum applied torque not more than 35 N/cm. The coronal fixture level was at the same level as the bone ridge.

In the mechanized insertion group, the fixture was removed from the box using a specific driver and inserted into the osteotomy site with light apical pressure. The torque was calibrated using a special rod (Surgic Pro S-MAX SG20 motor, NSK Co., Japan), and then adjusted to 35 N/cm, with 30 rpm speed. Insertion was continued until the coronal level of the fixture was the level of the osteotomy site.

In both groups, the cover screw was placed, and the flap was repositioned, and the incision was sutured with a silk suture. All of the suture materials were removed after 7 to 10 days of follow up. Prosthetic treatment was similarly performed on all patients after 3 months by performing the same mid crestal incision for healing abutment placement, and all the patients referred to the same experienced prosthodontist.

### Data acquisition and analysis

The marginal bone resorption in two groups was compared using three periapical radiographs taken by the paralleling technique immediately after the surgery, 3 months after the surgery, and 6 months after the surgery.

All the radiographs were taken in the same conditions by parallel technique and by using a film holder (XCP; Extended Cone Parallel, RinnCorp., Elgin, IL, USA) with the same equipment (63kVp, 8 mA, 0.08 s).

**Fig. 1:**
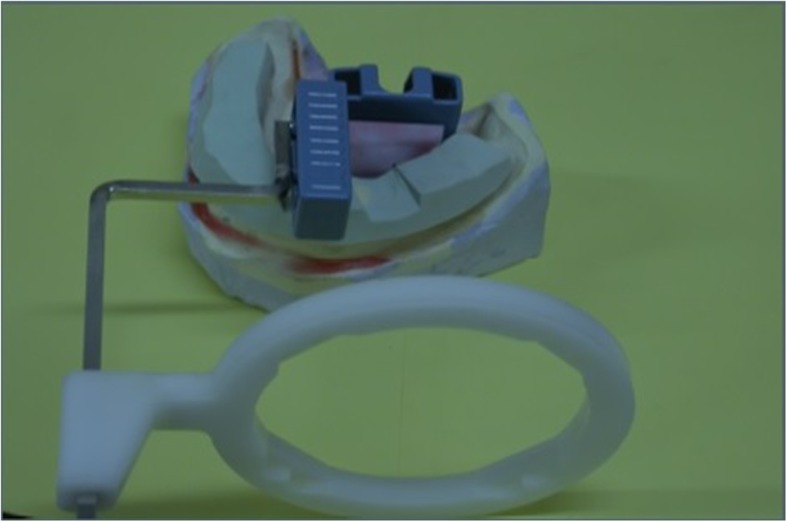
Film holder and putty index for the repeatable position for taking radiographs

The putty index was used to maintain the x-ray tube at the same horizontal angle and 10-cm tube-film distance. A path was prepared in the putty index for passage of the holding part of the film holder. The tube was adjusted according to the path that putty dictated to the film holder so that the position could be repeated in each session (Fig. 1).

The radiographs were all digitalized by a scanner and synchronized by subtraction method by using Adobe Photoshop CSS software (Adobe Systems Incorporated, San Jose, CA) so that the few differences from the tube angle change in radiography were modified. The constant implant length was counted as a basis for radiographic magnification measurements.

**Fig. 2:**
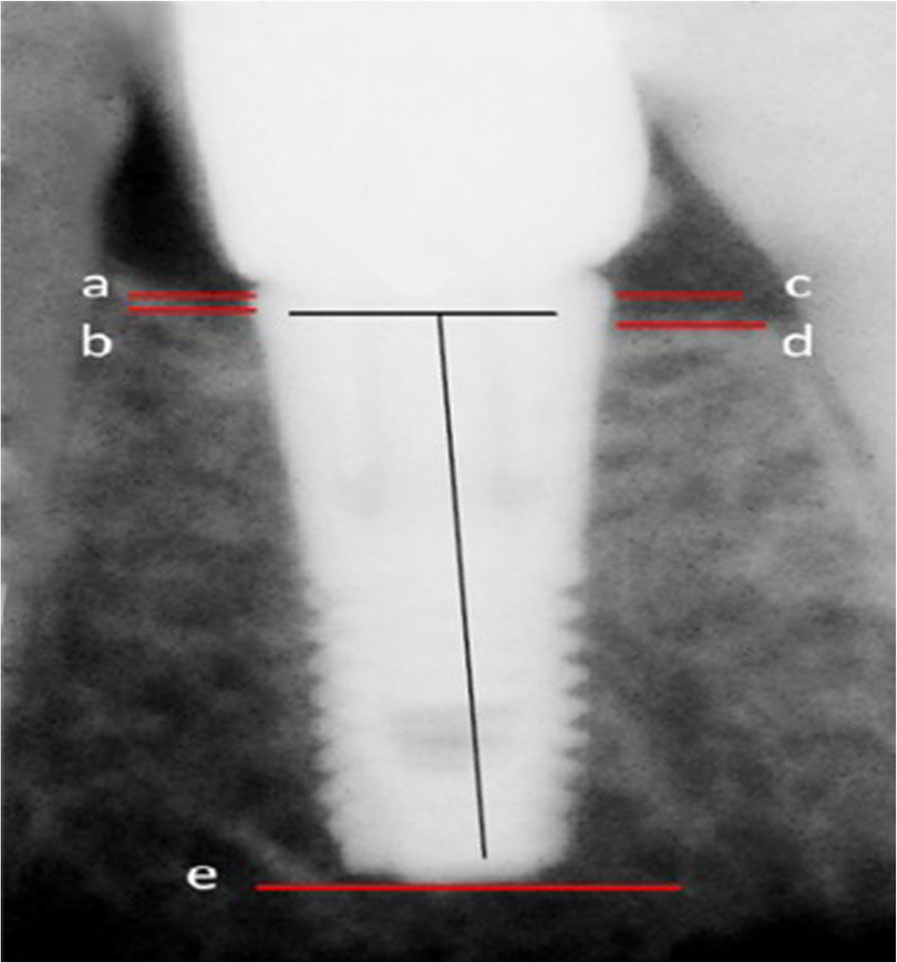
The mesial shoulder of the implant (**a**). The first contact area between the bone and implant at the mesial side (**b**). Distal shoulder of the implant (**c**). The first contact area between the bone and implant at the distal side (**d**)

For measuring the level of bone resorption, the shoulder of the implant was marked as a fixed index (Fig. 2: a and c points). The first contact area between the bone and implant in the mesial and distal area defines the level of resorption in the bone crest (Fig. 2: b and d points). The distance between the shoulder of the implant and bone crest was a standard for measuring the amount of bone resorption.

The resulting data were statistically analyzed by SPSS software, version 21. An independent t-test was used to compare the two groups regarding the average level of bone resorption (*p* < 0.05).

## Results

Intra-observer validation agreement was assessed by re-examination of 20% of data. The intraclass correlation coefficient was highly acceptable (ICC = 0.89). Kolmogorov-Smirnov test was used to assess the normality assumption for bone resorption. Because there was no significant deviation from normality, a parametric test (independent t-test) was used to compare the groups. Although the standard deviation was notably large in the manual group after 3 months, the Kolmogorov-Smirnov test did not show any violation from the normality assumption.

This randomized clinical trial randomly evaluated 273 patients (120 males and 153 females), aged 25–67 years. The implant insertion was manual in 167 patients (200 units) and mechanized in 106 patients (200 units). This study was performed on 273 cases randomly selected out of those referring to the oral and maxillofacial department. No premature cover screw exposure happened in this study group. The minimum bone resorption (0.44 ± 0.84 mm) was reported in the manual technique after 3 months, and the maximum (0.67 ± 0.19 mm) was observed in the mechanized technique after 6 months. (Table 1).

**Table 1:** Mean marginal bone loss (mm) using two dental implant insertion methods

Follow up periods	Groups	Number of patients(n)	Mean ± Standard deviation	*P*-value
Three months	Manual	200	0.44 ± 0.84	0.24
	Mechanized	200	0.51 ± 0.20	
Six months	Manual	200	0.59 ± 020	< 0.00
	Mechanised	200	0.67 ± 0.19	

The average bone resorption in manual method was 0.44 ± 0.84 mm after 3 months and 0.59 ± 0.20 mm after 6 months follow up, and for the mechanized method was 0.51 ± 0.20 mm after 3 months and 0.67 ± 0.19 after 6 months.

An independent t-test was run to compare the average bone resorption in the manual and mechanized techniques. At the end of the third month, there was no significant difference between the manual and mechanized technique regarding the average bone resorption after 3 months (*p* = 0.24). The same test was repeated 6 months later to compare the two groups regarding bone resorption. The results showing a significant difference between the two groups concerning the average implanted bone resorption after 6 months (*p* < 0.001, Table 1).

## Discussion

Considering the biologic and mechanical properties of the bone, the success of an implant treatment highly depends on preventing bone overload [28]. In other words, a portion of energy, which is applied during the implant insertion, can convert to heat. Since the bone hardly conducts the thermal energy, overheating leads to bone necrosis and resorption in the implant surrounding tissues [29].

The present study aimed to compare the different contributing factors during different implant insertion methods and the subsequent marginal bone resorption around dental implants, which were administered through manual and mechanized techniques. This comparison was made three and 6 months after the implant insertion. The results of this study demonstrated no significant difference between the manual and mechanized implant insertion techniques concerning the level of bone resorption in the mesial and distal surfaces of the implants after 3 months. However, by the end of the 6-month follow-up, bone resorption in the manual technique was lower than that in the mechanized insertion technique.

Bone quality is an essential factor in the amount of implant insertion torque. The applied torque is more critical for the mandible due to the higher density of the mandibular bone [30]. Wikenheiser et al. reported that the torque applied during the implant insertion was among the most critical factors that affected the degree of bone resorption. Accordingly, although higher torques increased the bone resorption, torque of 35 N/cm was required to achieve suitable stability [31]. Considering this point in the current study, the torque of 35 N/cm was considered optimal in both insertion methods. In different sites, the implants are inserted through a great variety of surgical procedures, drilling speed, applied force, cooling systems, type of the drill, as well as the time and depth of drilling. Berglundh et al. noticed that implant failure occurred far more in the maxilla than the mandible, and in posterior regions than the anterior parts. They attributed the failure to the quality of bones in different areas [32]. This factor was considered in the present study; hence, all of the implants in the posterior mandible were compared. However, if the surgeon believed that the bone was not of D2 or D3 bone quality, the patient was excluded from the study. The role of all the above-mentioned variables has been investigated in modifying the implant function; yet, the difference between the manual and mechanized insertion techniques was never studied before.

One of the studies about the differences between the manual and mechanized techniques by Novsak et al. focused on the use of orthodontic mini-implants via mechanized or manual methods. They inserted 120 mini implants through six different methods (three manual and three mechanized) into the ribs of a pig in laboratory conditions. It was observed that the manually-inserted implants showed higher stability and a lower level of bone resorption around them [33], which is similar to our results.

Implant manufacturing companies usually recommend mechanized drivers and handpieces for reaching sufficient insertion torque and ultimate clinical success. Misch et al. reported that in less dense bones (D2 and D3), handpieces are more appropriate for implant insertion and provide the maximum needed force [13]. In another study, Novsak et al. tested the general dentists’ ability to use manual drivers in an artificial area with limited access like the mouth. It was found that the use of manual ratchets was not practical in the posterior regions, because the dentist was not fully able to reach the needed torque and correctly inserted the implant into the target area [33]. In the present study, the site of insertion was the same in all cases (posterior of mandible). Although the mechanized insertion technique is straightforward, it is associated with the plausible decrease of the tactile sense and increase applied of force. An overload in the applied force might have negative consequences such as overheating, excessive pressure on the tissues, and bone necrosis.

Data analysis in this study showed that the level of bone resorption in the mesial and distal surfaces of the implants in the manual technique after three and 6 months was less than that in mechanized technique. This difference was statistically significant only in the six-months follow-up. However, the decrease in tactile sense control in mechanized technique can justify the possibility of overload in the applied force and its negative consequences such as overheating and excessive pressure on the tissue and bone, and higher bone resorption in mechanized technique after 6 months.

The limitations of the present study were that although we have attempted to minimize several factors that affect marginal bone loss using different insertion methods, controlling all of these factors is challenging. An oral and maxillofacial surgeon with extensive experience in implant dentistry performed all of the surgical procedures to minimize operator-dependent factors such as the surgical preparation of the implant site. Patient-dependant factors such as the quality of individual oral hygiene during the healing period were difficult to control, which can influence marginal bone loss. Every patient in this study was given postoperative oral hygiene instruction verbally and in writing, and this instruction was reinforced in every follow-up visit. Patients with inadequate oral hygiene were excluded to minimize marginal bone loss due to poor oral hygiene.

Drawing an absolute conclusion is still impossible due to the limited number of relevant studies, the nonexistence of buccal and lingual bone resorption measurement, high implant costs, lack of patient cooperation, and difficulty of long-term patient follow-up. Thus, further studies with more extended follow-up periods are recommended. Besides, modern imaging techniques such as cone-beam computed tomography are more appropriate for the evaluation of the implant surrounding the bones and provide more comprehensive data about all the surfaces.

## Conclusions

It seems that, by manual technique, long-term marginal bone loss around the implant fixture could be less than the mechanical method, but there was no significant difference between the manual and mechanized implant insertion techniques concerning the level of bone resorption in the mesial and distal surfaces of the implants after 3 months. However, after the six-month follow-up, the bone resorption in the manual technique was lower than that in the mechanized insertion technique.

## Data Availability

The datasets used and analyzed in this study are available on reasonable request from the authors (Saleh.khaqani@gmail.com, tavanafar@sums.ac.ir, s.tavanafar@gmail.com).
